# Bladder Carcinoma With Solitary Skull Metastasis: A Rare Presentation in a Middle-Aged Female

**DOI:** 10.7759/cureus.63171

**Published:** 2024-06-25

**Authors:** Manasa Suryadevara, Gaurav V Mishra, Pratapsingh Parihar, Ravishankar Patil, Anshul Sood, Nirja Thaker, Suhit Naseri, Shivani S Bothara, Rishabh Dhabalia

**Affiliations:** 1 Radiodiagnosis, Datta Meghe Institute of Higher Education and Research, Wardha, IND; 2 Pathology, Datta Meghe Institute of Higher Education and Research, Wardha, IND

**Keywords:** ct scan, solitary metastasis, urinary bladder carcinoma, skull invasion, skull bladder metastasis

## Abstract

Bladder carcinoma is a common malignant tumor of the urinary system, with the leading cause of death being the metastasis of cancer. It, however, is a rare malignancy in the Indian population with the incidence being higher in males compared to females. The most common sites of metastasis for bladder carcinoma are the peritoneum, liver, lung, pleura, lymph nodes, adrenals, intestine, and kidney. Metastasis to the heart and brain are rare. Only a few cases of bladder cancer metastasizing to the skull have been reported to date. Here in this article, we describe a female patient who presented with metastasis to the calvarium from bladder cancer before the identification of the original tumor.

## Introduction

Bladder carcinoma is a complex disorder, the 6th most common malignancy in the world [1]. The incidence of bladder carcinoma has been low in India, which is slowly rising due to smoking and exposure to certain chemicals like aniline and benzidine. Schistosomiasis, endometriosis, cystocele, and urinary obstruction are other risk factors for bladder cancer [2]. The occurrence of bladder carcinoma is higher in men compared to women, being more common in patients aged more than 55 years [3]. They are commonly urothelial carcinomas and, more commonly, not muscle invasive [1]. The patient commonly presents with hematuria and can be diagnosed by imaging, tumor resection, and cytology [4]. Computed tomography (CT) scan remains the primary imaging technique for the evaluation of bladder malignancy. There is also growing interest in advanced imaging techniques, like multiparametric magnetic resonance imaging (MRI), for local staging. Additionally, the recently established Vesicle Imaging Reporting and Data System (VI-RADS) aims to standardize imaging and reporting [1].

Metastasis of the tumor to the calvarium can occur in several cancers, most frequently originating from the prostate, breast, thyroid, and lung. Bladder carcinoma typically spreads to the bone marrow, liver, and lungs. However, skull metastasis from urothelial carcinoma of the urinary bladder (UCB) is rare, occurring in less than 1.0% of patients with this condition. When distant metastasis occurs, the prognosis becomes poor. A solid mass causing lytic lesions in the skull indicates metastasis and can present with a range of imaging findings. Metastasis to the skull can lead to numerous clinical symptoms, like local swelling, ulceration of skin, cosmetic issues, pain, bleeding, dural sinus compression, neurological deficits, and sinus thrombosis. Metastasis to the bone significantly deteriorates the prognosis for cancer patients due to the progression of the primary cancer or organ infiltration. If metastases to the cranium invade or overlie the dural venous sinus, resecting them surgically can offer effective palliation for symptomatic calvarial metastasis. Additionally, focal palliative radiotherapy can help relieve symptoms and prevent recurrence [5]. One of the key goals in staging the tumor is to determine whether it has invaded the muscle, as this significantly affects the prognosis and management. We present a patient who is a middle-aged woman with calvarial metastasis from bladder cancer.

## Case presentation

**Figure 1:**
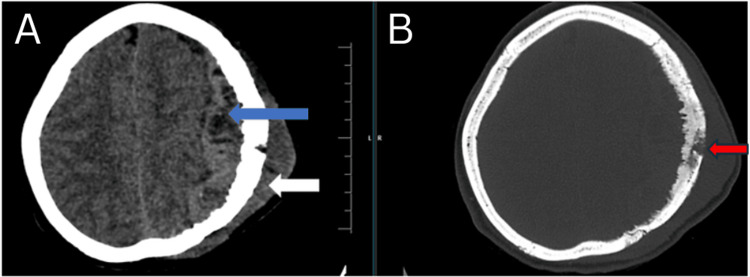
(A) Computed tomography brain plain axial section: brain window showing extra-calvarial (white arrow) and intra-calvarial, extra-axial (blue arrow) hyperdense lesion in the left frontoparietal region with areas of hypodensity within; (B) computed tomography brain plain axial section: bone window showing irregular destruction of the inner and outer table of the calvarium in the left frontoparietal region showing osteolysis (red arrow)

A middle-aged 40-year-old woman presented to our hospital (Acharya Vinobha Bhave Rural Hospital, Wardha, India) with a complaint of swelling in the left frontoparietal region, which has progressively increased in size for three months. The patient also complained of decreased urine output for the past two weeks. The patient denied a history of any nicotine abuse or chemical product exposure. A family history of malignancies was denied. The patient has been advised CT scan, which revealed irregular destruction of the inner and outer table of the left frontoparietal bone with hyperostosis and a few areas of hypodensity within, suggesting osteolysis. There is also evidence of adjacent extra- and intra-calvarial large hyperdense lesions (9.7 x 9 x 5.7 cm) with multiple areas of hypodensity and calcifications within, causing a mass effect in the form of effacement of sulcogyral spaces and left lateral ventricle and a significant midline shift of 15 mm toward the right side (Figure 1).

**Figure 2:**
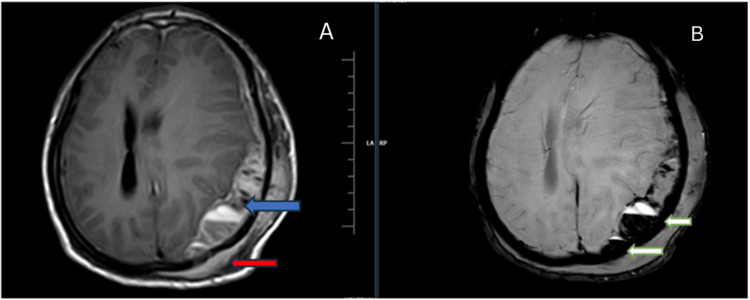
Magnetic resonance imaging brain axial section. (A) T1WI contrast sequence showing heterogeneously enhancing subdural (blue arrow) and extra-calvarial (red arrow) soft tissue lesion in the left frontoparietal region with erosive lysis of the bone; (B) SWI sequence showing fluid levels of blooming (white arrows) suggesting hemorrhage T1WI: T1-weighted image; SWI: susceptibility weighted imaging

The patient has been further advised contrast-enhanced magnetic resonance imaging (CE MRI) of the brain which showed the hyperosteotic erosive lysis of the bone with associated subdural and extra-calvarial soft tissue. The lesion is seen extending 22 mm into the subdural space, while the thickness of the extra-calvarial component measured 15 mm. Within the intracranial component, areas of acute subdural hemorrhage were noted with internal septations. The lesion appears heterogeneously intense on T1-weighted imaging (WI) with heterogenous enhancement on contrast administration (Figure 2).

**Figure 3:**
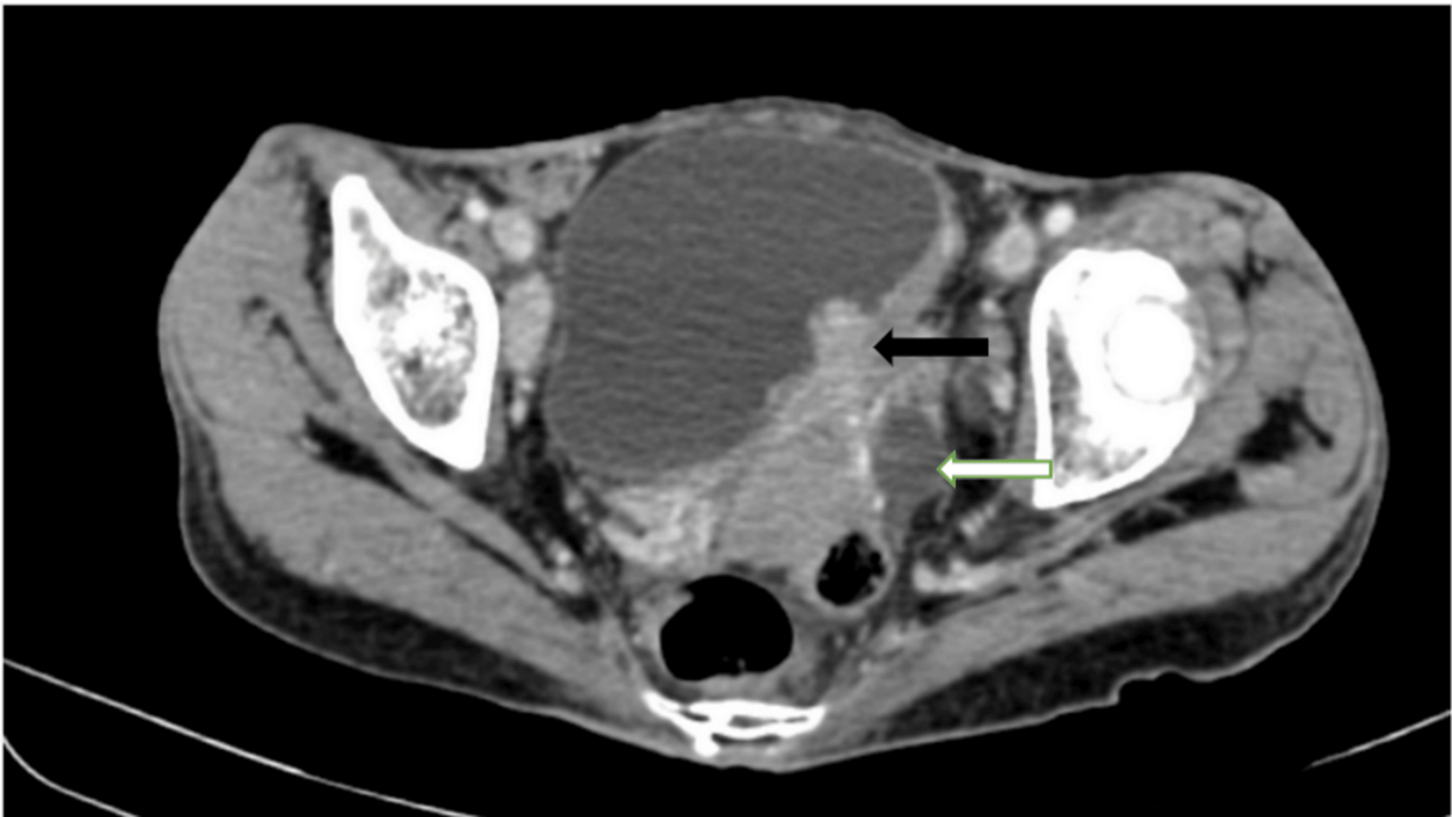
Contrast-enhanced computed tomography axial section shows heterogeneously enhancing polypoidal soft tissue lesion (black arrow) arising from the left lateral wall of the urinary bladder involving the left vesicoureteric junction causing upstream dilatation of the left ureter (white arrow)

**Figure 4:**
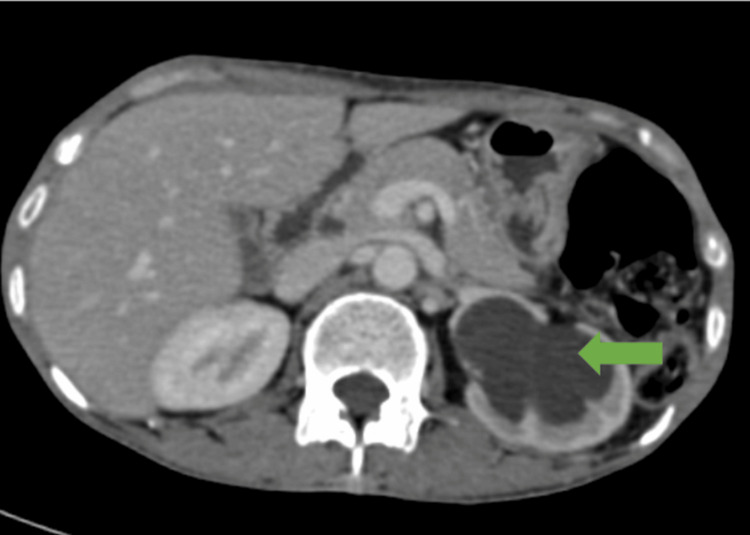
Contrast-enhanced computed tomography axial section shows dilatation of the left renal pelvis (green arrow) due to the obstruction of the left vesicoureteric junction by the mass lesion in the urinary bladder

Since the scans suggested metastasis, the patient, as a part of a workup to rule out the primary tumor, underwent contrast-enhanced computed tomography (CECT), which revealed an inhomogeneously enhancing polypoidal soft tissue lesion arising from the left lateral wall of the urinary bladder involving the left vesicoureteric junction causing upstream dilatation of the left ureter (Figure 3) and renal pelvis with cortical thinning (Figure 4).

**Figure 5:**
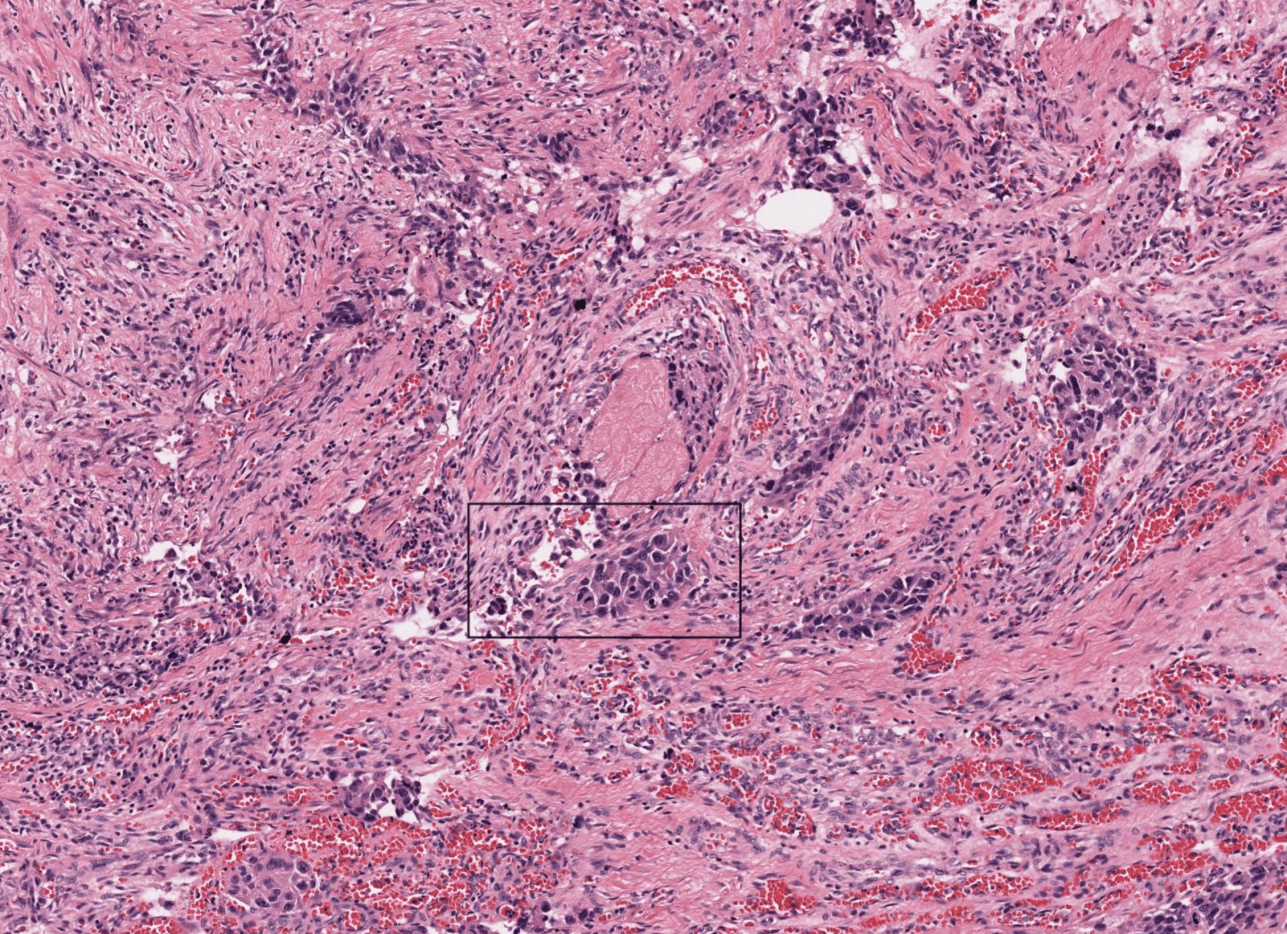
Hematoxylin and eosin, 20x section from the bladder mass shows nests, sheets, cords, or single cells invading the lamina propria with mixed architectural pattern (black box). Histopathological features are suggestive of invasive urothelial carcinoma

The patient has undergone transurethral resection of the bladder tumor (TURBT), and the specimen has been sent for biopsy. Histopathological examination showed nests, sheets, cords, or single cells invading the lamina propria with mixed architectural patterns suggestive of invasive urothelial carcinoma, a high-grade urothelial carcinoma (Figure 5).

The patient has been advised of chemoradiation and surgical resection as further management. However, the patient had monetary issues and hence did not wish to continue the treatment. So the patient has been discharged against medical advice.

## Discussion

Bladder carcinoma is a common malignancy involving the genitourinary system, and various risk factors have been associated with it. The peak incidence of the carcinoma is in the 6th decade of life. Risk factors include male gender, older age, personal or family history of bladder cancer, occupational exposure, smoking, radiation, chronic bladder irritation/infection, and the use of certain medications [6]. A patient can present with different symptoms; however, the most common presenting sign is painless hematuria. Bladder cancer can metastasize hematogenously to the bone and infiltrate the prostatic or vesical venous plexuses. The cancer can also spread to distant sites via the vertebral venous system, bypassing the major caval system [7].

The most common metastasis sites for bladder carcinoma are the bones, liver, lungs, peritoneum, and lymph nodes. Bladder carcinoma can commonly metastasize to the skeleton, with muscle-invasive urothelial carcinomas presenting with metastasis accounting for up to 27% [8]. The calvarium can be a site for tumor metastasis, as with any other bone in the body. The main sources of metastasis to the skull are the breast, prostate, and lung, responsible for over 70% of secondary tumors in the skull [9]. The calvaria comprises an inner table, an outer table, and bone marrow space in between. The calvarial metastases can usually involve all three layers. Lesions can occur in the calvarium's occipital, temporal, parietal, or frontal bones [10]. They are mostly asymptomatic and can cause a painless local swelling when they grow. Pain can occur if it involves the dura mater or periosteum, and symptoms of seizures, neurological deficits, or meningeal irritation can occur with brain compression, cranial nerves, or dural sinuses [11].

Patients with metastases to the calvarium often indicate an advanced disease. Only a few studies have emphasized the clinical manifestation of metastases to the calvarium. Their detection is challenging, particularly in patients without a known cancer. Sometimes, a solitary calvarial metastasis might be the sole indication of metastatic disease. In our patient, no significant complaints were present, pointing to the origin of the primary tumor apart from a swelling over the head [11]. CT scans are usually used to assess the extent and type of bony abnormalities. At the same time, MRI offers added data, such as multiplicity, early bone marrow involvement, and the anatomical relationship of the lesion to the brain and other structures in the cranium. Both the imaging modalities offer detailed morphological insights [9]. Surgical management is safe, being associated with low mortality, and is particularly suggested for lesions that are accessible and could lead to significant bone destruction, painful masses, dural infiltration, and neurological deficits, as well as to confirm a diagnosis or for solitary metastases [11].

## Conclusions

Bladder carcinoma is a rare occurrence in females as opposed to the common occurrence in old male patients. Calvarial metastasis from urothelial cancer is a sign of advanced disease and is a rare phenomenon presenting as the first indication of a primary tumor. MRI and CT scans allow for proper planning of the surgery, and positron emission tomography-computed tomography (PET-CT) stands as the most precise test for diagnosis of metastasis to the bone. Staging of the disease is crucial to ensure appropriate management.
